# Pauli-limit upper critical field of high-temperature superconductor La_1.84_Sr_0.16_CuO_4_

**DOI:** 10.1038/s41598-019-52973-1

**Published:** 2019-11-18

**Authors:** Daisuke Nakamura, Tadashi Adachi, Keisuke Omori, Yoji Koike, Shojiro Takeyama

**Affiliations:** 1 Institute for Solid State Physics, University of Tokyo, 5-1-5 Kashiwanoha, Kashiwa, Chiba 277-8581 Japan; 2 Department of Engineering and Applied Sciences, Sophia University, 7-1 Kioicho, Chiyoda-ku, Tokyo 102-8554 Japan; 3 Department of Applied Physics, Graduate School of Engineering, Tohoku University, 6-6-05 Aoba, Aramaki, Aoba-ku, Sendai, 980-8579 Japan

**Keywords:** Superconducting properties and materials, Phase transitions and critical phenomena

## Abstract

The upper critical field of a cuprate high-temperature superconductor, La_1.84_Sr_0.16_CuO_4_, was investigated by high-frequency self-resonant contactless electrical conductivity measurements in magnetic fields up to 102 T. An irreversible transition was observed at 85 T (*T* = 4.2 K), defined as the upper critical field. The temperature-dependent upper critical field was argued on the basis of the Werthamer-Helfand-Hohenberg theory. The Pauli-limiting pair-breaking process with a small contribution of the spin-orbit coupling explained the first-order phase transition exhibiting a hysteresis observed at low temperatures.

## Introduction

Magnetic field destroys superconductivity at the upper critical magnetic field, *B*_c2_, which provides information about the pair-breaking process in the superconducting state. In addition, a magnetic-field-induced normal state is realized even at low temperatures far below the superconducting transition temperature (*T*_c_), where the electronic state is unaffected by thermal fluctuation. Therefore, a novel quantum nature may be unveiled, which cannot be observed in the normal state in the absence of magnetic field. For example, the Fulde-Ferrell-Larkin-Ovchinnikov (FFLO) state^1–3^ and re-entrant superconductivity^4–6^ are theoretically suggested under high magnetic fields. This attracts attention of a high-field study on superconducting materials.

When a magnetic field is applied to a superconducting material, two independent mechanisms are considered to affect the pair-breaking process in the superconducting state: the orbital-limiting effect^7,8^ and Pauli paramagnetic (Pauli-limiting) effect^9,10^. In the case of the orbital-limiting effect, Cooper pair breaking is induced by the momentum, *eA*/ℏ*c*, where *A* is the vector potential, and eventually, the kinetic energy of supercurrent exceeds the superconducting gap energy. The upper critical field is recognized as the orbital-limiting field, *B*_orb_ = *ϕ*_0_/2π*ξ*^2^, which depends on the coherence length of Cooper pair, *ξ*. By contrast, in the case of the Pauli paramagnetic effect, the Zeeman splitting energy of electronic spin exceeds the superconducting gap energy, and singlet Cooper pair becomes energetically unstable. In this case, the upper critical field is recognized as the Pauli-limiting field, *B*_p_, where an irreversible first-order phase transition (FOT) is expected to be observed.

Cuprate high-temperature superconductors have a two-dimensional layered crystal structure, and electrical transport is confined in the CuO_2_ plane perpendicular to the *c*-axis. Therefore, the coherence length in the *ab*-plane is much longer than that along the *c*-axis, which induces an anisotropic pair-breaking mechanism depending on the direction of the magnetic fields applied to a crystal. The orbital-limiting effect is dominant under magnetic fields parallel to the *c*-axis (*B*//*c*). On the other hand, the Pauli-limiting effect is important under magnetic fields parallel to the CuO_2_ plane (*B*//*ab*), where *B*_orb_ becomes larger than *B*_p_. In general, *B*_c2_ in the *B*//*ab* configuration is larger than that in the *B*//*c* configuration.

Because *T*_c_ in cuprate superconductors largely depends on the carrier concentration, *B*_c2_ of the underdoped or overdoped materials can be suppressed below 100 T, which is sufficient to be generated by a non-destructive pulsed magnet. Following the pioneering work in 2007 on quantum oscillation above *B*_c2_ to characterize the Fermi surface^11^, numerous research studies about *B*_c2_ and quantum oscillations have been reported, particularly on underdoped YBa_2_Cu_3_O_7-*x*_ (YBCO)^12–25^. However, an optimally-doped YBCO (*T*_c_ ∼ 90 K) has a *B*_c2_ higher than 100 T, which can only be generated by a destructive pulsed magnet. For this material, extrapolation analysis has been conducted to evaluate *B*_c2_ (*T* = 0)^20,22,26^. Above 100 T, a high-field study of cuprate superconductors is rare owing to difficulties associated with the microsecond-order pulse duration time and the exploding nature of the destructive pulsed magnet. *B*_c2_ (*T* = 0 K) of YBCO has been evaluated to be 128 T and 240 T in the *B*//*c* and *B*//*ab* configurations^27^. In the above study, the magnetic field was generated by the electromagnetic flux compression technique^28^, where the measurement probe and sample were completely destroyed at the peak of a magnetic field pulse. This allowed the phase transition point at *B*_c2_ to be observable only in ascending magnetic fields. Therefore, the details of hysteresis cannot be discussed.

In this report, we focus on another type of cuprate superconductor, La_2−*x*_Sr_*x*_CuO_4_ (LSCO), whose *T*_c_ is approximately 40 K in the optimally-doped region (*x* ~ 0.16). Because its *T*_c_ is smaller than that of other typical cuprate superconductors, YBCO and Bi_2_Sr_2_CaCu_2_O_8+*x*_ (Bi2212, *T*_c_ ~ 90 K), *B*_c2_ (*T* = 0) of LSCO is easier to access. In an optimally-doped LSCO, the coherence length anisotropy takes a value between the corresponding values of YBCO and Bi2212 (YBCO: 5–7^29^, LSCO (*x* = 0.15): ~14^30^, Bi2212: 50–200^29^). Because two-dimensionality is one of the distinctive features of cuprate superconductors, the research on LSCO may become a linking bridge to understand the complete physics of cuprate superconductors.

In the *B*//*c* configuration, the transport property has been investigated up to 61 T^31–35^, and *B*_c2_ in the *B*//*c* configuration was found to be ~60 T at 4.2 K for an optimally-doped LSCO (*x* = 0.15)^34^. For an overdoped LSCO (*x* = 0.19), a recent high-field study up to 80 T has shown that the strength of the magnetoresistance (*dρ*/*dB*) does not depend on temperature below 25 K, which is different from the trend for conventional metals^36^. On the other hand, in the *B*//*ab* configuration, the measurement of *B*_c2_ in LSCO (*x* = 0.08, 0.14, and 0.18) has been performed only up to 17.5 T in the vicinity of *T*_c_^37^ and there has been no direct measurement of *B*_c2_ for an optimally-doped LSCO at low temperatures. In this study, we have directly observed *B*_c2_ of LSCO (*x* = 0.16) in the *B*//*ab* configuration by applying ultra-high magnetic fields up to 102 T.

**Figure 1:**
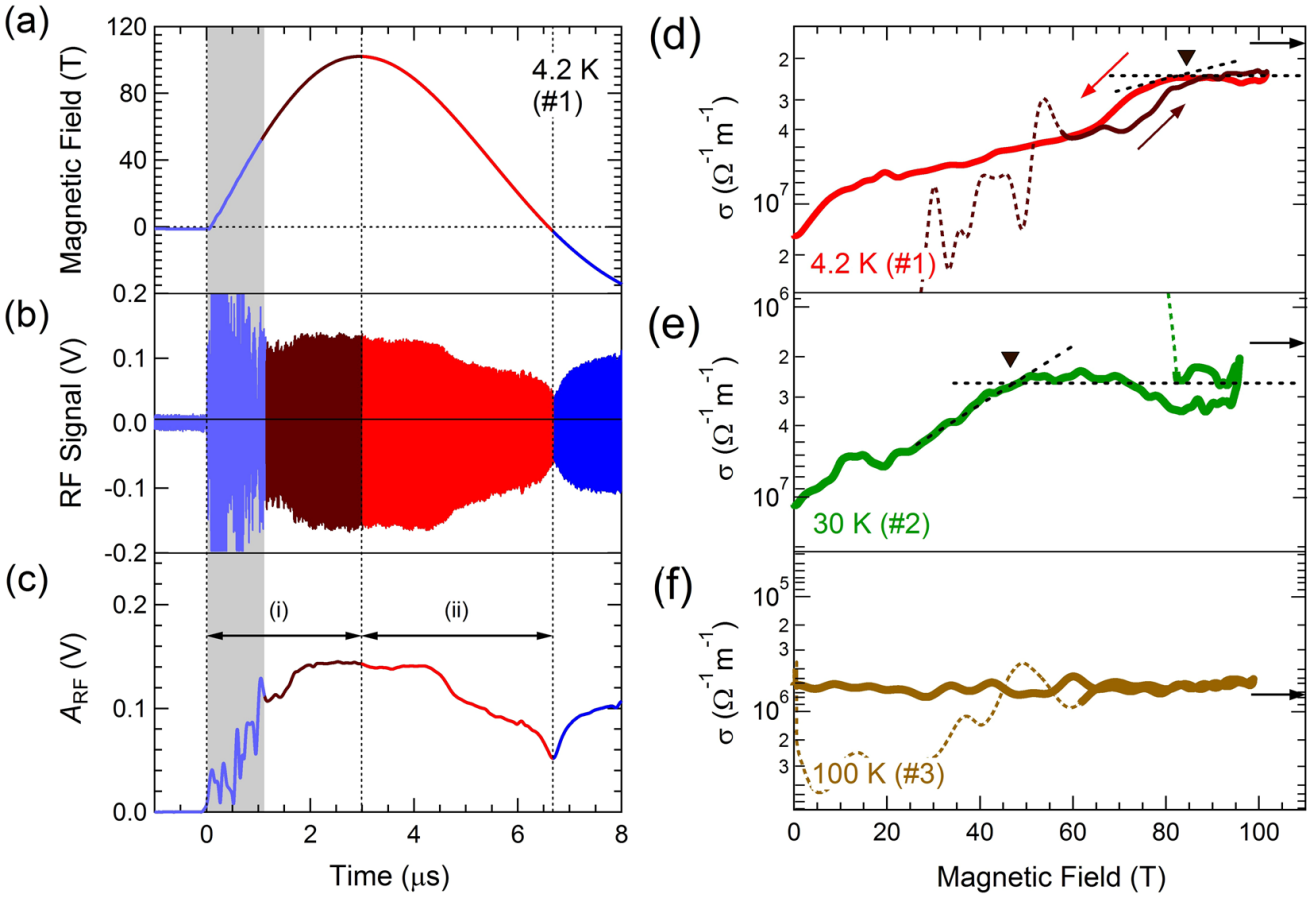
(**a**) Waveform of the magnetic field generated by the single-turn coil (STC) megagauss generator, (**b**) the RF probe signal, and (**c**) amplitude of (**b**) obtained in experiment #1 at 4.2 K. (**d–f**) Magnetic field dependence of the electrical conductivity at various temperatures. The down triangles denote *B*_c2_. The horizontal arrows indicate the values of the normal-state DC electrical conductivity at zero magnetic field. The colored broken lines show the data with electromagnetic discharging noise in the elevating slope of the magnetic field pulse. The panel (**d**) is based on the data published in Nakamura *et al*.^38^.

The results of the high-frequency electrical conductivity measurement using the self-resonant coil (SRC) at 4.2 K (#1) are displayed in Fig. 1(a–c), where the time evolution of the probe signal and its amplitude *A*_RF_ are presented with the waveform of the pulsed magnetic field. *A*_RF_(*t*) was analyzed in (i) an elevating and (ii) a descending slope of magnetic field pulse. The data in the first 1 μs in region (i) was excluded in the analysis (the hatched region in gray) owing to the condenser discharging large noises that disturb the probe signal.

The magnetic field dependence of the electrical conductivity, *σ*, at 4.2 K (#1), 30 K (#2), and above *T*_c_ (100 K, #3) is shown in Fig. 1(d–f). *A*_RF_ was converted to the value of *σ* by using a formula based on the electromagnetic analysis of the SRC described in ref.^38^ (also see Supplementary Fig. S3). The horizontal arrows in Fig. 1(d,e) indicate the electrical conductivity in the normal state at zero magnetic field (*σ*_DC_)^31^. The values of *σ*_DC_ below *T*_c_ were taken from *σ*_DC_ just above *T*_c_ (Fig. 1(d,e)). At 4.2 K (experiment #1, Fig. 1(d)), σ decreased with magnetic field and saturated at approximately 85 T (indicated by the down triangle), with evidence of a clear hysteresis. This type of magnetic field dependence was reproduced by an independent experiment (see Supplementary Fig. S4).

At 30 K (experiment #2), *σ*(*B*) decreased with magnetic field, and saturated at 46 T (down triangle). In the experiment far above *T*_c_ (100 K, #3), *σ*(*B*) exhibited a practically constant value in magnetic fields. This is consistent with a previous report of measurements up to 61 T^32^, stating that a change in the magnetoresistance above *T*_c_ is small for an optimally-doped LSCO.

*σ*(*B*) at 4.2 K (#1, Fig. 1(d)) continuously decreased up to *B*_c2_. In the *B*//*ab* configuration, the quantum flux called as the Josephson vortex selectively penetrates between the CuO_2_ layers. The decrease in *σ*(*B*) is considered to arise from the flow resistance of the Josephson vortex^39^. In addition, a magnetic field induces an energy shift of the quasiparticle excitation (Doppler effect^40^), which causes an increase in the density of the quasiparticle state for an anisotropic pairing symmetry, such as *d*-wave superconductor^41^. This effect also contributes to the decrease in *σ*. The kink structure around 10 T is considered to arise from a change in the Josephson vortex state. Various vortex states are theoretically suggested, which appear as a consequence of competition between the vortex-vortex interaction and the pinning force^42–45^. We note that *σ* in Fig. 1(d,e) does not diverge near zero magnetic field. The divergence in *σ* is difficult to observe by a contactless technique, because it has a finite measurable frequency range. In addition, a high-frequency probe signal induces a finite dissipation of the quasiparticle in the core of quantum flux, even in the superconducting state.

The hysteresis in *σ*(*B*) observed at 85 T is considered as an evidence of FOT at *B*_c2_. The melting transition of the vortex lattice cannot account for the hysteresis observed in Fig. 1(d). If this is the case, the high-field region above 85 T becomes the vortex liquid phase, where the superconducting order parameter remains, and *σ* continues to decrease with increasing magnetic field. However, our result in Fig. 1(d) shows that *σ* above 85 T is nearly constant within experimental error. Therefore, the high-field region above 85 T could be regarded as a magnetic-field-induced normal state, which arises after the Cooper pair breaking takes place completely.

**Figure 2:**
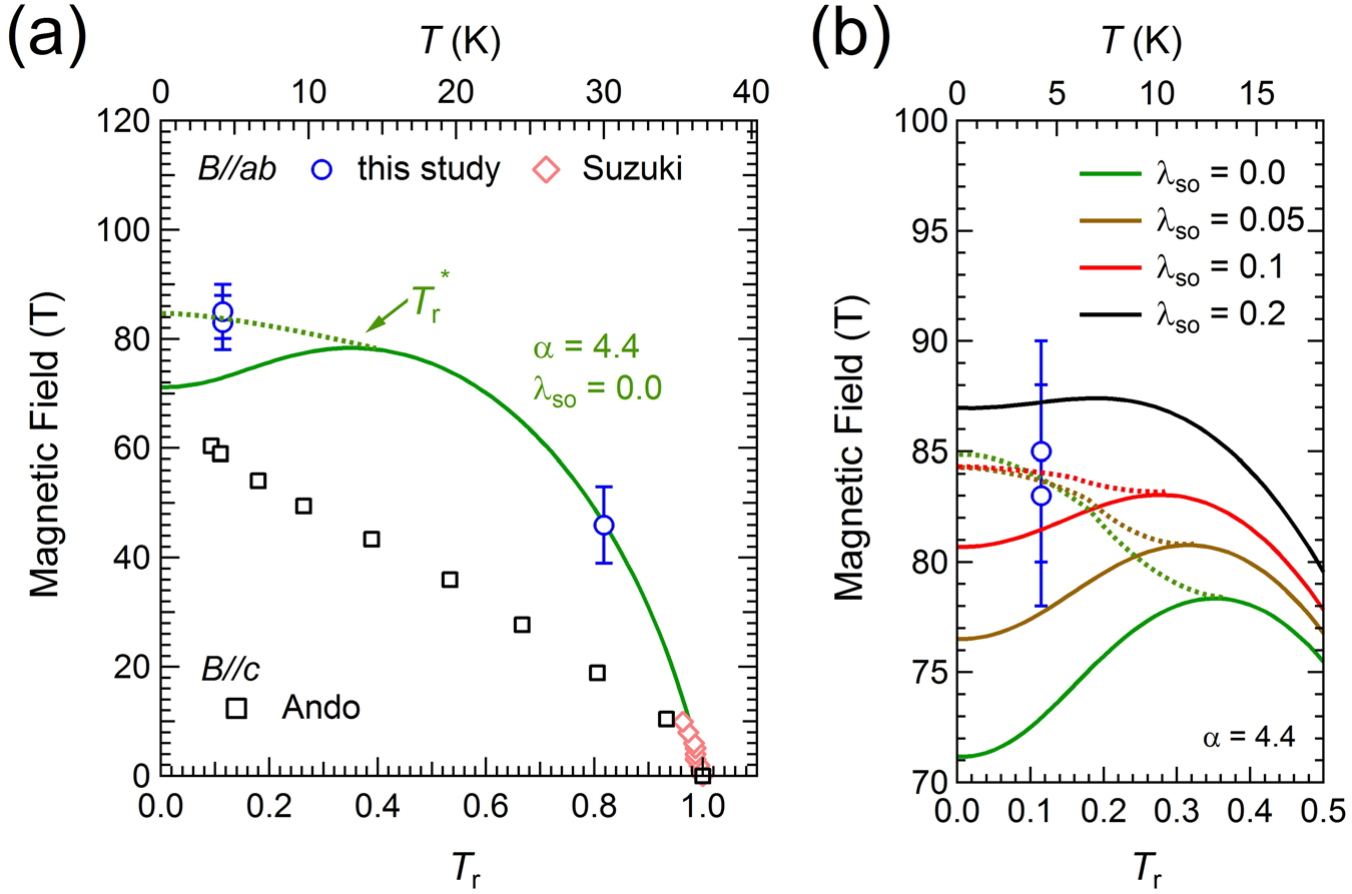
(**a**) *B*-*T* phase diagram of an optimally-doped LSCO. The open circles denote *B*_c2_ measured by using an STC magnet in this study, and the open diamonds (colored in pink) are *B*_c2_s near *T*_c_ reported in ref.^30^. The open squares are *B*_c2_ in the *B*//*c* configuration determined by Ando *et al*.^34^. The solid line shows the upper critical field of the WHH theory, *B*_WHH_. *T*_r_^*^ is the temperature at the local maxima of *B*_WHH_(*T*). The dotted line is a guide for the eye, indicating the critical field of the first-order transition, *B*_FOT_. (**b**) Enlarged plot of (**a**). Several *B*_WHH_ and *B*_FOT_ (guide) are displayed as solid and dotted lines, respectively.

Joule heating induced by the short pulse magnetic field cannot be a major cause of the hysteresis observed in *σ* (*B*). Typical phenomena caused by Joule heating can be represented in Fig. 2(b) of the previous report by Sekitani *et al*.^46^, in which *σ*(*B*) shows significant deviation in the entire region of ascending and descending magnetic fields. In order to confirm the effect of Joule heating on our sample, we have conducted similar contactless transport measurements by using long pulse magnet (Magnetic fields up to 55 T with a duration time 35 ms, indicating that *dB/dt* is reduced by four orders of magnitude. See Supplementary Fig. S5.), to see a good coincidence except a tiny difference of *B*_c2_ at 30 K. Therefore, we believe that the Joule heating effect is not significant in this study.

A superconducting-normal (S-N) transition is expected to be the FOT, when the Pauli paramagnetism causes the pair-breaking of superconductivity^9,10^. The Pauli-limiting field is expressed as *B*_p_  =  Δ_SC_/$\surd 2$ μ_B_, where μ_B_ is the Bohr magneton and Δ_SC_ is the superconducting gap energy. Given Δ_SC_ of LSCO as 2Δ_SC_ = 4.3 *k*_B_*T*_c_ evaluated from the ARPES measurement^47^, *B*_p_ is estimated to be 83 T in our sample, which is close to *B*_c2_ assigned in Fig. 1(d). It should be noted that the relationship, 2Δ_SC_ ~ 5 *k*_B_*T*_c_, has been confirmed for a number of cuprate superconductors^48^. The common relation held in LSCO implies that the superconducting energy scale in cuprate superconductors is governed by a universal mechanism.

The upper critical fields were plotted against temperature to display the magnetic field-temperature (*B*-*T*) phase diagram in Fig. 2(a). The lower abscissa in Fig. 2(a) is the reduced temperature, *T*_r_ = *T*/*T*_c_. The open circles are the *B*_c2_(*T*_r_) values obtained in this study in the *B*//*ab* configuration, and the error bars indicate an accuracy of the estimation of *B*_c2_ in the descending slope of the magnetic field pulse in Fig. 1(d,e). The open squares plot *B*_c2_ (*T*_r_) in the *B*//*c* configuration of LSCO (*x* = 0.15), as reported by Ando *et al*.^34^. It is noted in Fig. 2(a) that the anisotropy of *B*_c2_ is reduced at low temperatures, where the Pauli paramagnetic effect becomes a dominating factor. This tendency is in contrast to the orbital-limiting case. The *B*_c2_(*T*_r_) data just below *T*_c_ (open diamonds) determined by ref.^30^ are added to the plot in Fig. 2(a). We note that the carrier concentration (*x* = 0.15) of LSCO in refs^30,34^ is slightly different from that of our sample (*x* = 0.16). The *B*_c2_ (*T*_r_) values for LSCO with *x* = 0.15 and 0.16 are expected to practically coincide, because both enter into an optimally-doped region, where *T*_c_ takes a broad maximum in the electronic phase diagram.

A magnitude of the Pauli paramagnetic effect is described by the Maki parameter, *α*, which is defined as the ratio of the orbital-limiting field, *B*_orb_, and *B*_p_^49^,

1 $$\alpha =\sqrt{2}{B}_{orb}/{B}_{p} \sim 0.52758{(-\frac{d{B}_{c2}}{dT})}_{T={T}_{c}}$$

According to Maki’s theory, *α* is experimentally evaluated from the slope of *B*_c2_ close to *T*_c_. In LSCO, *dB*_c2_/*dT* was estimated to be 8.33 T/K at around *T*_c_, indicating *α* = 4.4. This value is compatible with those for other examples. The heavy fermion compound, CeCoIn_5_, is a typical Pauli-limiting superconductor^50^, and *α* is evaluated to be 4.6 in the *B*//*ab* configuration and 5.0 in the *B*//*c* configuration^51^. In CeCoIn_5_, a discernible hysteresis is observed to exhibit the FOT in the magnetization curve^52^.

*B*_c2_(*T*_r_) in Fig. 2(a) was analyzed by using the Werthamer-Helfand-Hohenberg (WHH) theory^8^, which considered both the Pauli paramagnetic effect and the spin-orbit interaction. The Maki parameter and the WHH theory have been used to analyze *B*_c2_(*T*) in high-temperature superconductors such as cuprates^27,46^ and iron-pnictides^53–56^. The temperature dependence of the upper critical field based on the WHH theory is described as follows^8^:

2 $$\mathrm{ln}\,\frac{1}{{T}_{r}}=\mathop{\sum }\limits_{\upsilon =-\infty }^{\infty }\{\frac{1}{|2\upsilon +1|}-{[|2\upsilon +1|+\frac{{B}_{r}}{{T}_{r}}+\frac{{(\alpha {B}_{r}/{T}_{r})}^{2}}{|2\upsilon +1|+({B}_{r}+{\lambda }_{so})/{T}_{r}}]}^{-1}\}$$

where the dimensionless magnetic field, *B*_r_ = 2*eB*_WHH_ *v*_F_^2^*τ* / (6π*k*_B_*T*_c_), is defined by using the upper critical field within the frameworks of the WHH theory, *B*_WHH_, Fermi velocity, *v*_F_, and carrier scattering time, *τ*. *λ*_SO_ is the spin-orbit coupling parameter, which provides the magnitude of the spin-flip scattering in material. In our analysis, the Fermi velocity and scattering time cannot be evaluated separately. Because the Maki parameter is described as $\alpha =3\hslash /2{m}_{e}{v}_{F}^{2}\tau$ in the WHH theory^8^, *v*_F_^2^*τ* is calculated to be 3.9 × 10^−5^ m^2^/s for *α* = 4.4. This is smaller than 5.8 × 10^−4^ m^2^/s evaluated from the results of the ARPES measurements; *v*_F_ = 2.9 × 10^5^ m/s^57^ and the inverse of mean free path 1/*v*_F_*τ* = 0.05 × 10^10^ m^−1^ ^58^. The difference in *v*_F_^2^*τ* may come from the simplification in the WHH theory, ignoring the Fermi surface anisotropy and strong electron-phonon coupling. In relation to this issue, we need an additional scaling factor *s* to fit *B*_c2_ by *B*_WHH_; *B*_r_ = 2*eB*_WHH_ *v*_F_^2^*τ s*/ (6π*k*_B_*T*_c_). Therefore, we calculated *B*_r_(*T*_r_) for parameters (*α*, *λ*_SO_), and then, determined *s* (~1.5) to fit *B*_c2_ = 46 T of the experiment #2.

In the case of (*α*, *λ*_SO_) = (0, 0), the pair-breaking is solely caused by the orbital-limiting effect (*B*_WHH_ = *B*_orb_). In this case, *B*_WHH_(*T* = 0) is evaluated to be 0.69*T*_c_(*dB*_c2_/*dT*)_*T*=*T*c_ = 211 T for a dirty superconductor^8^, which is much different from the experimental result. Using *α* = 4.4 determined by the *dB*_c2_/*dT* slope near *T*_c_, *B*_WHH_(*T*_r_) for (*α*, *λ*_SO_) = (4.4, 0.0) is presented as a solid curve in Fig. 2(a), which exhibits a local maximum at *T*_r_^*^ = 0.34. At low temperatures, *B*_c2_ are evidenced to deviate from *B*_WHH_. According to the WHH theory^8^, *B*_WHH_ represents the critical field of the second-order S-N transition. When the Pauli paramagnetic effect is dominant, *B*_WHH_ takes a local maximum at a finite temperature, *T*_r_^*^. The Pauli paramagnetic effect on the upper critical field has been described in detail by Maki and Tsuneto^10^. The FOT occurs at the upper critical field, *B*_FOT_ (>*B*_WHH_), below *T*_r_^*^. In this case, *B*_WHH_ below *T*_r_^*^ does not represent the phase transition point, but becomes the supercooling field. Accordingly, the hysteresis observed in *B*_c2_ at 4.2 K (*T*_r_ = 0.11) is a natural consequence when the upper critical field is dominantly determined by the Pauli paramagnetic effect. In Fig. 2(a), a dotted line was added as a guide for eyes, where the critical field becomes *B*_FOT_.

The spin-orbit coupling parameter *λ*_SO_ is also estimated in the following way. In Fig. 2(b), we show the enlarged plot of the *B*-*T* phase diagram at low temperatures. When we set *α* = 4.4, *B*_WHH_s for several values of *λ*_SO_ are plotted as solid curves. *B*_FOT_ (dotted guideline) is considered to locate above *B*_WHH_(*T*_r_^*^), because *B*_FOT_ has a negative slope at the low-temperature limit (*B*_FOT_ = (Δ_SC_^2^/2 −(π*T*)^2/3^)^0.5^)^10^. Therefore, the upper limit value of *λ*_SO_ is determined from the relation, *B*_WHH_(*T*_r_^*^) = 84. In this manner, the spin-orbit coupling parameter was estimated to be less than 0.1 as is notified from the plot in Fig. 2(b). *λ*_SO_ relates to the spin-flip scattering time *τ*_2_, *λ*_SO_ = 1/3π*T*_c_*τ*_2_^8^. The condition *λ*_SO_ < 0.1 indicates that *τ*_2_ > 1.4 × 10^−12^ s, which is far larger than the total scattering time *τ* = 6.9 × 10^−15^ s evaluated from the results of the ARPES measurements^57,58^. The magnitude relation between *τ* and *τ*_2_ is consistent with the assumption of the WHH theory, stating that the spin-flip scattering process is infrequent compared with the spin-independent scattering process (*τ* < < *τ*_2_)^8^.

**Figure 3:**
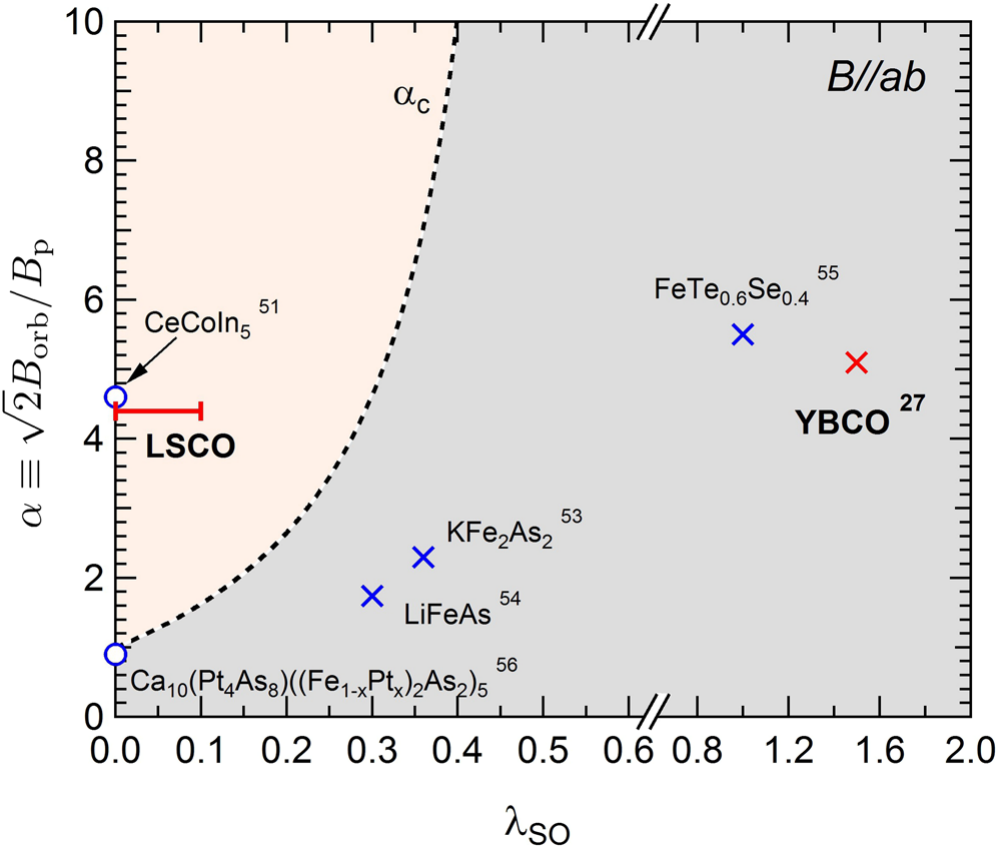
*α* -* λ*_SO_ phase diagram. In the pink-colored region above *α*_c_, the first-order phase transition at *B*_c2_ is predicted by the WHH theory. The cross symbols denote (*α*, *λ*_SO_) parameters for materials with a lack of hysteresis at *B*_c2_^27,53–55^. The open circles represent the values of *α* for materials with a hysteresis observed at *B*_c2_^51,56^. We note that the parameter, *λ*_SO_, is not considered in the analysis in refs^51,56^.

The spin-orbit coupling suppresses the Pauli paramagnetic effect, because the spin-flip scattering induces an effective reduction of the Zeeman splitting of the energy levels of electrons in the singlet Cooper pair. In the *α*-*λ*_SO_ diagram shown in Fig. 3, a FOT occurs at *B*_c2_ when *α* > *α*_c_ (pink-colored region)^8^, where

$${\alpha }_{c}=\frac{1+1.589({\lambda }_{so}/0.5139)}{1-({\lambda }_{so}/0.5139)}\cdot $$

The parameter (*α*, *λ*_SO_) of LSCO (*x* = 0.16) is located inside the pink-colored region. In contrast, *B*_c2_(*T*) of another typical cuprate superconductor, YBCO, is well fitted by the WHH parameter (*α*, *λ*_SO_) = (5.1, 1.5) in the *B*//*ab* configuration^27^, which is outside of the pink-colored region in Fig. 3. Therefore, from the discussion above, it could be inferred that the S-N transition in YBCO can hardly be a FOT at low temperatures. In Fig. 3, (*α*, *λ*_SO_) of other superconductors are also shown as blue cross symbols (no hysteresis observed at *B*_c2_^53–55^) and blue open circles (hysteresis observed at *B*_c2_^51,56^). We note that the parameter *λ*_SO_ is not included in the analysis in refs^51,56^.

In cuprate superconductors, the magnitude of the spin-orbit coupling is considered to depend on the buckling in the CuO_2_ plane. When the inversion symmetry perpendicular to the CuO_2_ plane breaks, a Rashba-type spin-orbit coupling occurs^59^. Among the cuprate superconductors, the buckling in YBCO is the largest, where the Cu-O-Cu angle is approximately 165 deg^60,61^. Actually, the strong spin-orbit coupling in YBCO was evidenced by the high-field quantum oscillation of side-frequencies in the nodal direction of the *d*-wave order parameter^62^. Contrastingly, in LSCO, the Cu-O-Cu angle is approximately 177 deg^63^, indicating that the CuO_2_ plane is practically flat. Therefore, the spin-orbit coupling is suggested to be much smaller than that in YBCO, which is consistent in our study, as shown in Fig. 3. In terms of the spin-flip scattering time, *τ*_2_ of YBCO can be evaluated to be 3.8 × 10^−14^ s, from *T*_c_ ~ 90 K and *λ*_SO_ = 1.5^27^_._ This value is considerably smaller than that of LSCO.

From the above discussion, FOT at *B*_c2_ induced by the Pauli paramagnetic effect is only realized in cuprate superconductors when the strict conditions, (i) the *B*//*ab* configuration and (ii) a small buckling in the CuO_2_ plane, are satisfied. In this respect, LSCO can be regarded as a rare case among cuprate superconductors, in which *B*_c2_ is studied as the FOT. A comparison with a typical Pauli-limiting superconductor, CeCoIn_5_, which exhibits an exotic high-field phase such as the FFLO state in the vicinity of *B*_c2_, might impose a novel guideline to reveal the magnetic-field-induced superconducting properties.

## Conclusion

In summary, the electrical conductivity measurements using the SRC method were performed for a cuprate high-temperature superconductor, La_1.84_Sr_0.16_CuO_4_, under ultra-high magnetic fields up to 102 T using a single-turn coil megagauss generator. The upper critical field was investigated in the *B*//*ab* configuration, where a first-order phase transition was observed at low temperature. Comparison with the WHH theory indicated that both a large Maki parameter and a small amount of the spin-orbit coupling parameter were necessary to explain the temperature dependence of *B*_c2_ and the Pauli-limiting first-order phase transition. These conditions were only realized for the *B*//*ab* configuration and a small buckling of the CuO_2_ plane in La_1.84_Sr_0.16_CuO_4_.

### Experimental methods

The optimally-doped LSCO (*x* = 0.16, *T*_c_ = 36.7 K) single crystal was synthesized by the traveling-solvent floating-zone method^64^. The concentration of Sr ion was determined by inductively-coupled-plasma optical-emission-spectrometry^65^. The samples were cut to a thin tabular shape parallel to the *ab*-plane, whose dimension was 1 × 1 × 0.05–0.07 mm^3^.

Magnetic fields up to 102 T were generated by a vertical single-turn coil (STC) megagauss generator^66^. The duration of the pulsed magnetic field with a damped sinusoidal shape is 6 μs. Because the destruction of the magnet coil takes place in outward direction, a measurement sample is intact after the magnetic field generation. Therefore, a hysteresis phenomenon, if any, would be observable on the elevating and descending slopes of the magnetic field pulse. The magnetic field was applied along the *ab*-plane of LSCO sample (*B*//*ab*). In the STC magnet, a liquid helium cryostat was set, enabling the measurements at temperatures down to 2 K^67^.

The in-plane electrical conductivity of LSCO sample was measured by the contactless self-resonant coil method^38,68^. A home-made planar probe coil worked by itself as an LCR resonant circuit (self-resonant coil, hereafter called as SRC). The *ab*-plane of sample was mounted on the SRC, and radio-frequency (RF) current was applied to the SRC as a probe signal. The experimental set-up and measurement conditions are described in Supplementary Materials. When the electrical conductivity of a sample changes, the resonant spectrum of an SRC is affected by the electromagnetic coupling between a sample and an SRC. Therefore, when an RF current closely tuned to the resonant frequency (*f*_res_, ~800 MHz) was applied to an SRC, the return RF voltage responded sensitively to changes in the electrical conductivity of the sample, *σ*. The amplitude of the return RF voltage, *A*_RF_, was monitored by a high-resolution (12 bit) digital oscilloscope.

## Supplementary information

Supplementary Materials

## Data Availability

The datasets generated during and/or analyzed during the current research are available from the corresponding author on reasonable request.
