# Supplementary material for: Pauli-limit upper critical field of high-temperature superconductor La1.84Sr0.16CuO4
Source: Sci Rep. 2019 Nov 18;9:16949. doi: 10.1038/s41598-019-52973-1 (PMC6861275; doi:10.1038/s41598-019-52973-1)
Supplement: Supplementary file 1 — Supplementary Materials [file 41598_2019_52973_MOESM1_ESM.pdf]

Supplementary Materials of "Pauli-limit upper critical field of high-temperature superconductor $La_{1.84}Sr_{0.16}CuO_4$"

Daisuke Nakamura[1], Tadashi Adachi[2], Keisuke Omori[3], Yoji Koike[3] and Shojiro Takeyama[1]

[1]*Institute for Solid State Physics, University of Tokyo, 5-1-5 Kashiwanoha, Kashiwa, Chiba 277-8581, Japan*
[2]*Department of Engineering and Applied Sciences, Sophia University, 7-1 Kioicho, Chiyoda-ku, Tokyo 102-8554, Japan*
[3]*Department of Applied Physics, Graduate School of Engineering, Tohoku University, 6-6-05 Aoba, Aramaki, Aoba-ku, Sendai 980-8579, Japan*

A. High-field experiments using self-resonant coil method

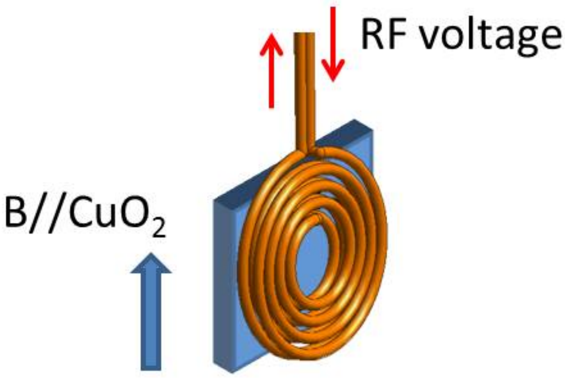

Fig. S1. Schematic view of the self-resonant coil and the measurement sample.

The contactless electrical conductivity measurement was performed by the self-resonant coil (SRC) method [1, 2]. The self-resonant coil is a home-made spiral-type coil with 8-10 turns. The coil inductance, wire resistance, and stray capacitance between wires form the resonant circuit, whose resonant frequency is around 800 MHz. The *ab*-plane of measurement sample was placed on the plane of the self-resonant coil as shown in Fig. S1, and fixed by an epoxy. The magnetic field was applied parallel to the *ab*-plane, for reducing the Joule heating effect by the Eddy current. The experimental conditions of #1-#4 are described in Table SI.

Exp.	Sample	Thickness	T	B_{max}
#1	#A	50 μm	4.2 K	102 T
#2	#A	50 μm	30 K	90 T
#3	#A	50 μm	100 K	100 T
#4	#B	70 μm	4.2 K	102 T

Table SI. The parameter of experiments. The sample, thickness of sample, measurement temperature, and maximum magnetic field intensity generated by the STC magnet are listed.

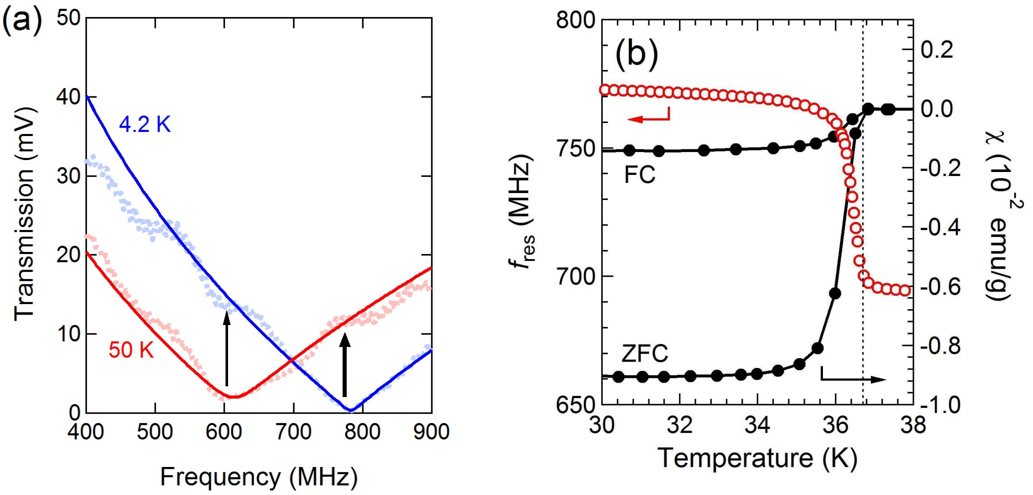

Fig. S2: (a) Resonant spectra of the SRC with LSCO (x = 0.16). The arrows indicate frequencies of injected probe signal (thin arrow: #3, thick arrow: #1, #2). (b) Temperature dependence of the resonant frequency of SRC (open symbols) and the magnetic susceptibility of LSCO (x = 0.16) (closed symbols). The dotted line indicates T_c.

Figure S2(a) shows the resonant spectra of the SRC mounted with LSCO sample (markers). The resonant spectra could be fitted by using the impedance parameters of an LCR resonant circuit (solid curves). f_{res} of SRC (open symbols in Fig. S2(b)) started to decrease rapidly at T_c = 36.7 K, which was determined from the magnetic susceptibility (closed symbols), with increasing temperature. This plot demonstrates a sensitive response of the resonant spectrum to σ of LSCO sample. Most of the electrical conductivity measurements were carried out with the frequency 774 MHz (thick upward arrow in Fig. S2(a)), close to f_{res} in the superconducting state. At 774 MHz, suppression of the superconductivity by the magnetic field is expected to result in a substantial increase of A_{RF}. The frequency 604 MHz was chosen as the RF probe signal for the measurement at 100 K (#3), so as to improve the measurement sensitivity (thin upward arrow in Fig. S2(a)).

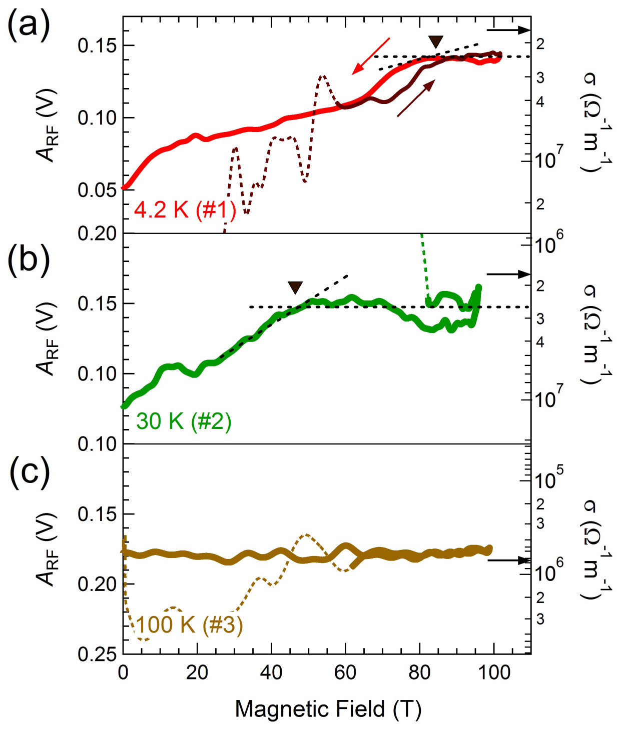

Fig. S3. (a)-(c) Magnetic field dependence of the amplitude of the RF probe signal and electrical conductivity at various temperatures. The down triangles denote B_{c2}. The horizontal arrows indicate the values of the normal-state DC electrical conductivity at zero magnetic field. The colored broken lines show the data with electromagnetic discharging noise in the elevating slope of the magnetic field pulse. The panel (a) is based on the data published in Nakamura *et al*[2]..

The amplitude of the probe signal, A_{RF}, was converted to the value of the electrical conductivity, σ by using a formula based on the electromagnetic analysis of the SRC described in Supplementary Ref. 2. The magnetic field dependence of σ and A_{RF} at 4.2 K (#1), 30 K (#2), and above T_c (100 K, #3) is shown in Figs. S3(a)-S3(c). We note that σ increases with A_{RF} only in Fig. S3(c) owing to the difference in frequency of the probe signal for the measurement at 100 K as shown in Supplementary Fig. S2(a).

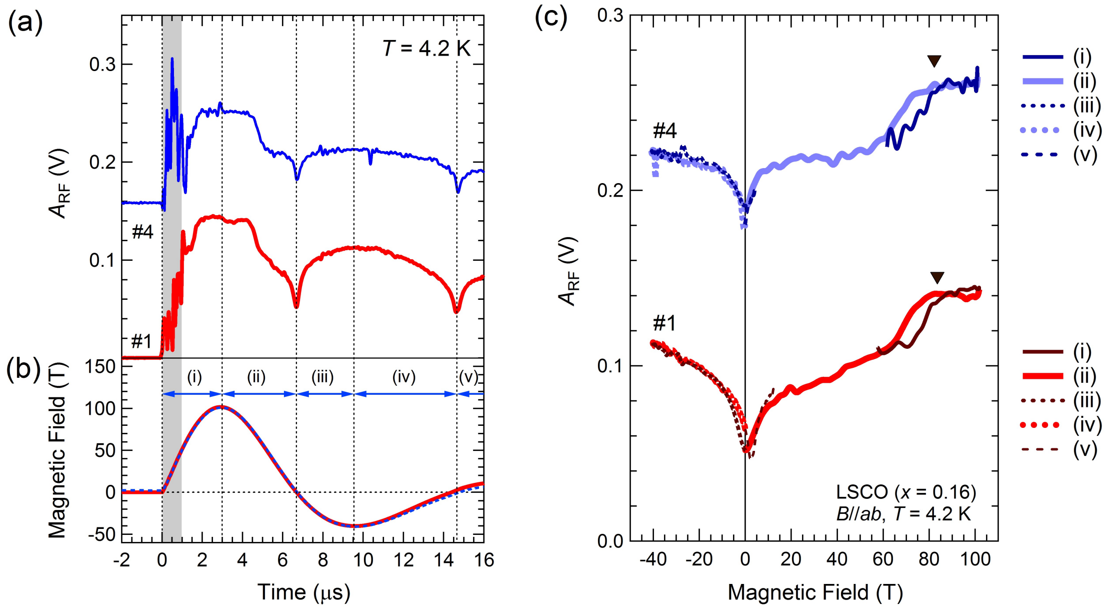

Fig. S4. (a) Time dependence of the amplitude of probe signal and (b) waveforms of magnetic field pulse in experiments #1 and #4, performed at 4.2 K. (c) Magnetic field dependence of A_{RF}. Down triangles indicate B_{c2}, above which A_{RF} saturates. The different types of curve indicate the time regions (i)-(v) shown in (b).

The magnetic field dependence of A_{RF} in experiments #1 and #4 performed at 4.2 K were compared in Fig. S4. As shown in Fig. S4(c), all $A_{RF}(B)$ traces develop in a similar manner with a clear hysteresis at B_{c2}. Therefore, obtained results in this study are proven to be intrinsic.

B. Supporting data obtained by using non-destructive pulsed magnet

For the comparison, the transport measurement up to 55 T was performed using the non-destructive pulsed magnet with longer pulse duration time of 35 ms. The contactless tunnel diode oscillator (TDO) method [3, 4] similar to the self-resonant coil method was employed to detect the electrical conductivity of LSCO sample. Figure S5 shows the amplitude of the TDO signal, A_{TDO}, measured at 30 K (below T_c) and 38 K (the normal state just above T_c). At 30 K, A_{TDO} gradually increased with magnetic field, and the saturation was observed. B_{c2} was defined to be 44.5 T (upward arrow in Fig. S5), where $A_{TDO}(B)$ reached to 90 % of A_{TDO} in the normal state. This value was consistent with the result shown in Fig. 2(e). Because the Joule heating effect in a longer-pulse conventional pulsed magnets is less than that in the single-turn coil magnet, consistent value of $B_{c2}(T = 30\ \mathrm{K})$ in both measurements indicates that our results is free from the issue of the Joule heating effect.

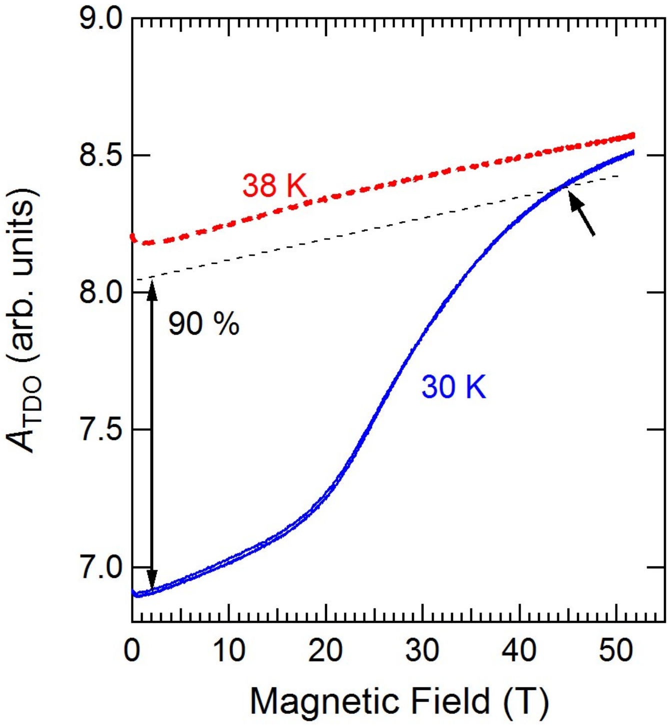

Fig. S5. Magnetic field dependence of the amplitude of TDO signal measured at 30 K (solid curve) and 38 K (dashed curve). The upward arrow indicates B_{c2}, defined by the 90 % value of A_{TDO} in the normal state (thin dashed curve).

Supplementary References

[1] M. M. Altarawneh, Rev. Sci. Instrum. **83**, 096102 (2012).

[2] D. Nakamura, M. M. Altarawneh, and S. Takeyama, Meas. Sci. Technol. **29**, 035901 (2018).

[3] T. Coffey *et al.*, Rev. Sci. Instrum. **71**, 4600 (2000).

[4] E. Ohmichi *et al.*, Rev. Sci. Instrum. **75**, 2094 (2004)
